# Consensus statement for cancer patients requiring intensive care support

**DOI:** 10.1007/s00277-018-3312-y

**Published:** 2018-04-27

**Authors:** M. G. Kiehl, G. Beutel, B. Böll, D. Buchheidt, R. Forkert, V. Fuhrmann, P. Knöbl, M. Kochanek, F. Kroschinsky, P. La Rosée, T. Liebregts, C. Lück, U. Olgemoeller, E. Schalk, A. Shimabukuro-Vornhagen, W. R. Sperr, T. Staudinger, M. von Bergwelt Baildon, P. Wohlfarth, V. Zeremski, P. Schellongowski, Consensus of the German Society of Hematology and Medical Oncology (DGHO), Austrian Society of Hematology and Oncology (OeGHO), German Society for Medical Intensive Care Medicine and Emergency Medicine (DGIIN), and Austrian Society of Medical and General Intensive Care and Emergency Medicine (ÖGIAIN)

**Affiliations:** 1 Department of Internal Medicine I, Clinic Frankfurt/Oder GmbH, Müllroser Chaussee 7, 15236 Frankfurt (Oder), Germany; 2 Hannover Medical School (MHH) Clinic for Hematology, Coagulation, Oncology and Stem Cell Transplantation, Carl-Neuberg-Straße 1, 30625 Hannover, Germany; 3 Department of Internal Medicine I, University Hospital, Kerpener Str. 62, 50937 Cologne, Germany; 4 III. Medical Clinic, Medical Faculty Mannheim, Theodor-Kutzer-Ufer 1-3, 68167 Mannheim, Germany; 5 Johanniter-Hospital, Johanniterstr. 3-5, 53113 Bonn, Germany; 6 Clinic for Intensive Care Medicine, University Hamburg, Martinistr. 52, 20246 Hamburg, Germany; 7 Department of Medicine I, Medical University of Vienna, Waehringer Guertel 18-20, 1090 Vienna, Austria; 8 Department of Internal Medicine I, University Hospital, Fetschertstr. 74, 01307 Dresden, Germany; 9 Department of Internal Medicine III, Schwarzwald-Baar-Klinikum, Klinikstr. 11, 78052 Villingen-Schwenningen, Germany; 10 Clinic for Stem Cell Transplantation, University Hospital Essen, Hufelandstr. 55, 45147 Essen, Germany; 11 Department of Cardiology and Pulmonary Medicine, University Hospital, Robert-Koch-Str. 40, 37075 Göttingen, Germany; 12 Department of Hematology and Oncology, University Hospital, Leipziger Str. 44, 39120 Magdeburg, Germany

**Keywords:** Hematological malignancy, Oncological malignancy, Critical illness, Intensive care treatment, Mechanical ventilation, ICU, ICU admission

## Abstract

This consensus statement is directed to intensivists, hematologists, and oncologists caring for critically ill cancer patients and focuses on the management of these patients.

**Electronic supplementary material:**

The online version of this article (10.1007/s00277-018-3312-y) contains supplementary material, which is available to authorized users.

## Introduction and background

Cancer is the second leading cause of death worldwide. According to recent publications, the incidence of neoplasms is expected to increase requiring more health care resources. Appropriate allocation of resources for cancer prevention, early diagnosis, as well as curative and palliative care remain of particular importance. This requires a detailed knowledge of the local burden of cancer [1].

In patients with hematological or oncological malignancies, the disease itself or intensive therapy may result in critical illness in a substantial number of patients. In 1999, the American College of Critical Care Medicine stated that patients with hematological or metastasized oncological malignancies are poor candidates for ICU admission with a mortality rate of up to 90%. Thus, refusal of ICU admission of these patients was the common procedure (Task Force of the American College of Critical Care Medicine, Society of Critical Care Medicine, 1999 [2]). However, within the last 25 years, cancer and ICU treatment clearly improved. As shown in recent reports, hospital mortality decreased to less than 40% in these critically ill patients with hematologic malignancies [3]. Thus, criteria for ICU admission have to be adjusted. Aim of this consensus statement is to provide a review of the recent literature and the development of practical recommendations to hematologists, oncologists, and intensive care specialists. Due to a series of practice-changing investigations on proper ventilation techniques in cancer patients with acute respiratory failure, we put a special emphasis on discussing this topic.

## Methods

The consensus group was selected to represent the expertise in the field of critical care in cancer patients in Germany and Austria. Experts were sent by the German Society of Hematology and Medical Oncology (DGHO), the Austrian Society of Hematology and Oncology (OeGHO), the German Society of Internal Intensive and Emergency Medicine (DGIIN), and the Austrian Society of Medical and General Intensive Care and Emergency Medicine (ÖGIAIN). All authors are actively supporting the “Intensive Care Medicine in Hematologic and Oncologic Patients (iCHOP)” Initiative (info@ichop.eu).

Consensus statements are based on an in-depth PubMed literature retrieval of English as well as German language literature with last access February 01, 2017. For medical heading terms (MeSH) identifying potential relevant literature, please see Appendix. Seven working groups (a: ICU transfer criteria and goals of therapy, b: respiratory failure, c: end of live decisions, d: stem cell transplant patients, e: infections, f: coagulation, and g: miscellaneous) prepared the draft proposals in a first round of consensus development. Working groups decided on references with relevant impact on guideline development, and every group member had to approve the draft guideline subchapter. Finally, the consensus statements were approved by the assembly of the members on March 2nd and 3rd 2017 in Vienna, Austria, a meeting hosted and sponsored by the OeGHO. All statements and manuscript wording underwent voting of the consensus assembly.

This guideline follows the structure and definitions of the ESCMID Guideline on Candida diseases [4–6]. It is in accordance with the GRADE and AGREE [7, 8] (Table 1).

**Table 1:** Strength of the recommendation and quality of evidence

Strength	Recommendation
A	Strong recommendation for use
B	Moderate recommendation for use
C	Marginal recommendation for use
D	Recommendation against use
Quality of evidence	
I	Evidence from at least 1 properly designed randomized, controlled trial
II^a^	Evidence from at least 1 well-designed clinical trial, without randomization; from cohort or case-controlled analytic studies (preferably from > 1 center); from multiple time series; or from dramatic results of uncontrolled experiments
III	Evidence from opinions of respected authorities, based on clinical experience, descriptive case studies

^a^Added index: _*r*_ : meta-analysis (or systematic review of RCT); _*t*_ : transferred evidence, i.e., results from different patients “cohorts” or similar immune status situation; _*h*_ : comparator group: historical control; _*u*_ : uncontrolled trials; _*a*_ : published abstract (presented at an international symposium or meeting)

All recommendations are given in boldface type.

## Prognosis and ICU admission criteria

### Whom to admit to the ICU/eligibility and goals of therapy

#### Full-code ICU management (without limitations of ICU resources) should be offered to all critically ill cancer patients if long-term survival may be compatible with the general prognosis of the underlying malignancy (A-IIu)

This statement, of course, presumes that the acute condition may be reversed by ICU measures, and that age and comorbidities of patients do not contradict such an approach. Typically, full-code management applies to patients with curative therapeutic options, those in remission of their malignancy, as well as to patients in whom cure is not likely but the expected life-span is substantial [3, 9]. It has been suggested in an earlier consensus that an assumed prognosis of 1 year may be used as cutoff for clinical decision making with regard to full-code status [10]. The authors of this manuscript believe this number is arbitrary and may be regarded as basis for individual considerations only.

#### Patients with poor performance status not eligible for further anti-cancer therapy, dying patients, as well as those rejecting critical care treatment should not be admitted to the ICU in general (A-III)

#### For patients not fulfilling the above-described criteria for full-code ICU management or ICU refusal, time-limited ICU trials or pre-defined do-not-escalate decisions (e.g., do-not-intubate or do-not-attempt-resuscitation) may be adequate options (B-IIu)

In a prospective trial on patients with *intermediary* prognosis (non-palliative, non-bedridden, non-full-code patients), those with worsening organ failure scores after days 5 of ICU therapy had a high risk of dying. The authors suggested to offer these patients a full-code ICU trial with re-evaluation of the goals of therapy after at least 6 days [9]. We would like to point out that almost all patients who died after day 5 had treatment-limitation decisions. Therefore, it remains unknown what their course would have been in case of further full-code management. Furthermore, data on cancer patients with *poor* prognosis suggest that the optimal duration of time-limited ICU trials seems to differ largely between patients with solid tumors and hematologic malignancies [11]. Thus, proposing a fixed interval from admission to re-evaluation of the goals of ICU therapy in patients undergoing time-limited ICU trials may not be generally appropriate.

### When to admit to the ICU, indications and screening for organ dysfunction

Several observational investigations on cancer patients with septic complications and respiratory failure indicate that early ICU admissions and early interventions, respectively, are associated with improved survival [3, 12–16]. Thus, early identification of patients at risk for critical deterioration seems crucial. Severity of illness scores can only be used for describing groups of patients and should, therefore, not be used in individual clinical decision making with regard to ICU admissions or prognostication.

#### Cancer patients should be considered for ICU admission in case of manifest or incipient acute organ dysfunction(s) (A-IIu)

#### Cancer ward patients at risk should be screened for the presence of sepsis daily (A-IIt)

#### Cancer ward patients at risk should be screened for the presence of acute organ dysfunction(s) daily (A-IIu)

### Recommendations for local structures and educational matters

Mortality of ICU cancer patients has markedly dropped over the last decades, presumably due to improved general ICU management, improved diagnostic and therapeutic strategies for several cancer-specific situations, as well as refined admission criteria [10, 17, 18]. Yet, prognostication of cancer patients and adequate patient selection remain far from perfect [19].

#### Centers should establish local admission criteria for critically ill cancer patients as well as standardized admission procedures, such as joint assessment by cancer specialists and intensivists, if available (A-III)

A recent Brazilian investigation showed an independent correlation between daily joint rounds of cancer specialists and intensivists as well as the number of protocols with lower mortality and more efficient resource use [20]. This is supported by Nassar and coworker stating that regular meetings may reduce possible conflicts between intensivists and oncologists [21]*.*

#### Centers should establish daily joint rounds of cancer specialists and intensivists as well as standard-operating procedures for the management of frequent medical conditions of critically ill cancer patients (A-IIu)

Although a recent study by Soares and colleagues failed to identify ICU volume as a prognostic marker for ICU survival [20], the majority of available studies demonstrate a significant association of case volume and ICU mortality in cancer patients with septic shock or acute respiratory failure [22–24]. This effect might be linked to expertise and specific processes of care. However, some of the available observational studies may not have accounted for potential confounders related to ICU organization, structure, and process of care. Ultimately recognizing and developing “critical care of cancer patients” as a medical subspecialty may optimally serve the needs of affected patients [25]. Nevertheless, due to the fast-growing demand for critical care for cancer patients, the major practical challenge is how to provide high-quality and affordable care for all patients. Treating all critically ill cancer patients regardless of the underlying condition in specialized centers may not be feasible after all. Therefore, managing some of the rather common cancer-specific ICU problems, such as neutropenic sepsis, should become part of routine expertise for all intensive care specialists. However, in more specific situations, such as chemotherapy administration in critically ill patients with aggressive hematologic malignancies or caring for ICU patients after allogeneic stem cell transplant, transfer to specialized centers should be discussed.

#### Centers should establish joint continuous medical education of cancer specialists and intensivists (A-III)

#### Basic concepts of the care of critically ill cancer patients should be included into the curricula of both cancer specialists and intensivists (A-III)

All previous statements are summarized in Table 2.

**Table 2:** Admission to ICU, eligibility, and aims of therapy

	Intention	Clinical situation/intervention	SoR/QoE	Reference
I.	Defining ICU eligibility criteria and goals of therapy	Full-code ICU management in all critically ill cancer patients with prospect of long-term survival	A-IIu	[3]
II.	Defining ICU eligibility criteria and goals of therapy	ICU refusal in patients with poor performance status not eligible for further anti-cancer therapy, dying patients, as well as those rejecting critical care	A-III	
III.	Defining ICU eligibility criteria and goals of therapy	Time-limited ICU trial and/or do-not-escalate decisions in patients neither fulfilling full code nor refusal criteria	B-IIu	[9, 11]
IV.	Reducing mortality; indications for ICU admission	Early admission of patients with manifest or incipient acute organ dysfunction(s) to the ICU	A-IIu	[3, 12–16]
V.	Reducing mortality; early identification of ICU candidates	Daily sepsis screening in cancer ward patients at risk	A-IIt	[26, 27, 28]
VI.	Reducing mortality; early identification of ICU candidates	Daily screening for acute organ dysfunctions in cancer ward patients at risk	A-IIu	
VII.	Facilitating ICU admission decisions	Local ICU admission criteria and joint assessment by cancer specialists and intensivists	A-III	
VIII.	Reducing mortality; local structures	Establish daily rounds of cancer specialists and intensivists	A-IIu	[20]
IX.	Reducing mortality; local structures	Establish standard-operating procedures for frequent medical conditions of critically ill ICU cancer patients	A-IIu	[20]
X.	Reducing mortality; local structures	Admit critically ill cancer patients to experienced ICUs	A-IIu	[19, 20]
XI.	Continuous medical education	Establish joint continuous medical education of cancer specialist and intensivists	A-III	
XII.	Basic medical education/training	Include basic concepts on critically ill cancer patients into cancer specialists’ and intensivists’ curricula	A-III	

## Acute respiratory failure (ARF)

### Diagnostic work-up

With respect to the critical prognosis of ARF in cancer patients, especially in patients with ARF of unknown origin [29, 30], diagnostic procedures are of major clinical importance. A systematic diagnostic work-up may lead to a (high-probability) diagnosis in approximately 70–80% of patients with ARF [31, 32]. However, diagnostic procedures should not cause a delay in the start of adequate ARF therapy including prompt antibiotic treatment (modification) in the case of a (suspected) infection (A-III).

The diagnostic strategy should encompass a comprehensive analysis of the clinical course of the underlying malignancy, including data on mechanisms of immunosuppression, antineoplastic and antimicrobial treatment, and prophylaxis [31, 33].

#### Independent of the clinical presentation, chest computed tomography is recommended (A-IIt)

#### Bronchoscopy and a BAL-based diagnostic work-up including a broad range of both culture- and non-culture-based diagnostic methods should be added to noninvasive tests depending on pretest probabilities of clinical etiologies (if clinically feasible early after ICU admission and without causing clinical worsening) (A-I)

Table 3 provides a summary of diagnostic procedures in cancer patients with ARF.

**Table 3:** Invasive and noninvasive diagnostic procedures in ARF

Blood cultures	
Multislice or high-resolution computed tomography (CT) scan of the lungs (in most cases without contrast media), (MRI of the lungs, if a pulmonary CT scan is not feasible)	
Echocardiography (cardiac status)	
Sputum examination for	Bacteria Fungi Mycobacteria
Induced sputum	*Pneumocystis jiroveci*
Nasopharyngeal aspirates	RSV, influenza
Polymerase chain reaction blood test for	Herpesviridae Cytomegalovirus Epstein-Barr virus
Circulating *Aspergillus* galactomannan	
Serologic tests for	*Chlamydia pneumoniae* *Mycoplasma pneumoniae* *Legionella pneumophila*
Urine antigen for	*Legionella pneumophila* *Streptococcus pneumoniae*
BAL (samples should include by default)	• Cytospin preparation including Giemsa stain for cytological diagnostics and Gram stain • Bacteriological cultures (quantitative or semi-quantitative) including culture media to detect *Legionella* spp., mycobacteria and fungi • Calcofluor white or equivalent stain (assessment of fungi) • (quantitative, if possible) PCR for *Pneumocystis jirovecii* • direct immunofluorescence test for *Pneumocystis jirovecii* • *Aspergillu*s antigen (Galactomannan ELISA) • *Mycobacterium tuberculosis* PCR, atypical mycobacteria
BAL (optional)	• PCR for cytomegalovirus, respiratory syncytial virus, influenza A/B virus, parainfluenza virus, human metapneumovirus, adenovirus, varicella zoster virus, and *Pneumocystis jirovecii* (quantitative) [34] • *Aspergillus* antigen (Galactomannan ELISA; ODI (optical density index): 1.0) Panfungal or *Aspergillus*/mucormycetes PCR
Transbronchial biopsies	Not recommended in general in febrile neutropenic and/or thrombocytopenic patients as a first line procedure.

Adapted and modified from Azoulay et al., Am J Respir Crit Care Med 2010 [31]

### Ventilatory strategies

Noninvasive ventilation (NIV) remains the first-line standard of care in acute exacerbate chronic pulmonary disease (AECOPD) or acute cardiogenic pulmonary edema [35]. However, the role of NIV in hypoxic ARF is far less well documented. While a historic single-center randomized controlled trial (RCT) suggested superiority of NIV over conventional oxygen therapy in immunocompromised (mainly hematologic) patients with regard to intubation and survival rates [36], successive (mainly observational) studies have led to conflicting results. The 2015 “*Clinical Practice Guidelines on noninvasive mechanical ventilation in ARF*” on behalf of the German Society of Pneumology and Ventilatory Medicine acknowledge that NIV can be attempted to avoid intubation in immunosuppressed (including hemato-oncologic) patients. In this regard, it is of particular importance to respect common contraindications and termination, i.e., intubation criteria [35]. However, the respective guideline could not account for several meanwhile published investigations: First, a meta-analysis on 2380 mainly hematologic patients revealed that NIV as initial ventilator strategy was associated with lower mortality, whereas this finding is a mere association and cannot be interpreted as proof of principle. Importantly, 61% (range 40 to 78) of patients experienced NIV failure with secondary intubation, which itself was associated with increased mortality [37]. Second, in a large multicenter observational investigation in 1004 cancer patients with ARDS, NIV failure occurred in 71% of patients and was, again, independently associated with mortality, while mortality of ARDS patients undergoing IMV per se had decreased to 52% in recent years [38]. Finally, a recent large multicenter landmark RCT compared the use of NIV to conventional oxygen in immunocompromised (mainly hematologic) patients with hypoxic ARF (acute cardiogenic pulmonary edema or hypercapnia, i.e., PaCO_2_ > 50 mmHg excluded, pre-specified intubation criteria [39]). The trial did not show any clinical benefits or increased harms associated with NIV. However, the lower than expected mortality rate in the oxygen only group limited the power to detect significant differences, and a higher proportion of patients in the NIV group received high flow nasal oxygen intermittently, which may have limited the demonstrable effects of NIV. Risk factors for NIV failure in cancer patients are depicted in Table 4.

**Table 4:** Risk factors for NIV failure in cancer patients with acute respiratory failure

Prior to NIV	Vasopressor need Multiple organ failure Airway involvement by malignancy Acute respiratory distress syndrome Unknown etiology of ARF Delayed onset of ARF
During NIV	Patient not tolerating NIV No improvement of ABG within 6 h Respiratory rate > 30/min NIV dependency ≥ 3 days Clinical or respiratory deterioration Unknown etiology of ARF

Adopted and modified from Soares et al., Critical Care Clinics 2010 [40]

*NIV*, noninvasive ventilation; *ARF*, acute respiratory failure; *ABG*, arterial blood gas

High flow nasal oxygen (HFNO) has recently been shown to be associated with reduced intubation (PaO_2_/FiO_2_ < 200) and mortality (PaO_2_/FiO_2_ < 300) rates in elderly patients with pneumonia and hypoxic ARF when compared to conventional oxygen therapy or NIV [41]. This effect was also observed in the subset of immunocompromised patients [42]. However, given conflicting data, the applicability of these findings in cancer patients, and specifically in those presenting with other etiologies than pneumonia, remains to be demonstrated [43, 44].

#### In case a NIV or HFNO trial is initiated in cancer patients with hypoxic ARF, common contraindications and/or the occurrence of pre-specified intubation criteria should lead to intubation and invasive mechanical ventilation without delay (A-IIu)

#### Given the considerable failure rates of NIV and HFNO in hypoxic ARF and the lack of sufficient data on the safety of such therapies in regular wards, NIV and HFNO should not be attempted in this indication on a regular ward (B-III)

### End-of-life, palliative care

Principles in palliative care and end-of-life (EOL) management are not different in cancer patients compared to critically ill patients without a malignancy as underlying disease (A-IIt). However, (i) patient’s wish, (ii) progressive (multi-) organ failure despite full-code ICU management and no chance of reversibility, or (iii) rapid progress of cancer disease without further treatment options have to be kept in mind and could change the treatment decision or treatment goals [45]. These issues have to be discussed between oncologists and the intensive care team in an interdisciplinary manner. In cancer patients admitted to an ICU, current treatment state and estimated prognosis may be unknown to the ICU team. Therefore, consultation of an oncologist is highly recommended to provide valid prognostic information (A-III).

Intensive care admissions often represent an additional burden for patients and families in situations without option for cure. Good communication is essential to allow patients and families to meet their preferences regarding EOL. According to the literature, too many EOL decisions are made too late [46]. Early integration of an interdisciplinary palliative care team for hospitalized patients with life-limiting disease leads to fewer ICU admissions (A-I) [47]. Furthermore, integration of pro-active palliative care on ICUs, including palliative care rounds, leads to a shorter length of stay on ICU or in the hospital (A-IIt) [48, 49].

To improve quality of EOL care in critically ill patients regarding important clinical questions, i.e., preparing for withdrawal, assessment and drug management of distress, discontinuation of treatment and monitoring, or dignity conserving care [50], the authors refer to published consensus recommendations [51, 52].

## Special situations

### Anticancer therapy during ICU

In some cases, for example pulmonary involvement due to high grade non-Hodgkin lymphoma, hyperleukocytosis, etc., despite ICU treatment initiation or completion of anticancer chemotherapy, immunochemotherapy or radiotherapy is necessary to improve patient’s clinical situation. Such some decision needs intensive discussions between the responsible hemato-oncologist and intensivist. Again, we recommend joint assessments by cancer specialists and intensivists and that basic concepts of the care of critically ill cancer patients should be included into the curricula of both cancer specialists and intensivists.

### Prevention and treatment of infections in critically ill cancer patients

Published guidelines on prevention, diagnosis, and treatment of infections in cancer patients do not specifically address critically ill cancer patients. In addition, few studies and current guidelines on infections in critically ill patients specifically address the particular requirements in the treatment of cancer patients (Table 5).

**Table 5:** Recommendations on prevention, diagnosis, and treatment of infections in cancer patients

	Intention	Clinical situation/Intervention	SoR/QoE	Reference
I.	Reducing mortality	Treatment in close collaboration of intensivist with cancer specialist and ID specialist	AIIt	[53, 54]
II.	Prevention of infections	Continuation of prophylactic antiviral, antibacterial and antifungal treatment upon ICU-admission, unless switch to therapeutic treatment or excessive organ toxicity	AIIt	[55, 56, 57]
III.	Prevention of infections	Immunization only according to specific guidelines for patients with cancer	BIII	[58]
IV.	Treatment	Treatment of critically ill patients with infections including neutropenic sepsis according to current guidelines for the treatment of sepsis and septic shock	AIIt	[59]
V.	Anti-infective treatment	Anti-infective treatment according to recommendations for treatment of bacterial, fungal and viral infections in cancer patients	AIIr	[60–64]
VI.	Reducing mortality	Recommendation against routine use of GCSF in neutropenic patients with pulmonary infiltrates	AIIr	[65–67]
VII	Reducing mortality	Use of GCSF only in selected cancer patients according to current recommendations.	AIIt	[68]

Resources on prevention and treatment of infections in cancer patients, which might be largely applicable to critically ill cancer patients, are guidelines on:

- Management of neutropenic patients in the intensive care unit [69]
- Prophylaxis of viral [57], bacterial [55], and fungal [56] infections including *Pneumocystis jirovecii* pneumonia [55]
- Unexplained fever in neutropenic patients [60] (new version in progress)
- Diagnosis of invasive fungal infections [70] (new version in progress)
- Treatment of invasive fungal infections [61] and *Pneumocystis jirovecii* pneumonia [62]
- Diagnosis and antimicrobial therapy of lung infiltrates in febrile neutropenic patients [63]
- Prophylaxis and treatment of infections in recipients of autologous [71] (new version in progress) and allogeneic stem cell transplant [26]
- Community-acquired respiratory viral infections [72]
- Management of central venous catheter-related infections [73]
- Gastrointestinal complications including neutropenic enterocolitis [74] (new version in progress).
- Infections of the central nervous system in patients with hematological disorders [75]
- The Surviving Sepsis Campaign (SSC) Guidelines for Management of Sepsis and Septic Shock recently published [76]. The guidelines published in 2014 on sepsis in neutropenic patients might be less appropriate since they are based on the 2012-ssc-guidelines [59].
- IDSA guidelines on immunization in patients with cancer [58]; guidelines on immunization by the Infectious Diseases Working Party (AGIHO) of the German Society of Hematology and Medical Oncology (DGHO) are under progress.
- Prophylaxis of infectious complications with colony-stimulating factors in adult cancer patients undergoing chemotherapy [68].

## Allogeneic stem cell transplant setting

### Criteria for ICU admission

The prognosis of allogeneic hematopoietic stem cell transplant (HSCT) patients admitted to an ICU has been significantly improved in the last years [77]. It is important to note that physicians of the primary hospital have to contact the patient’s transplant center immediately in case of critical illness. Allogeneic HSCT patients with early ICU admission or single organ dysfunction benefit from intensive care. However, patients with uncontrolled or refractory graft-versus-host disease (GvHD) should not be intubated for acute respiratory failure (A-III) [10, 78]. Thus, it is important to contact immediately patient’s transplant center.

For further information regarding isolation procedures, specific monitoring, as well as typical complications, please see supplement.

### Coagulation

Venous thromboembolism (VTE), including deep-vein thrombosis and pulmonary embolism, represents a major cause of morbidity and mortality in cancer patients. An increased tumor burden results in a higher risk for VTE with the highest risk in patients receiving systemic chemotherapy or being hospitalized on surgical and medical floors.

Although there are no validated data on the risk assessment in cancer patients on the ICU, according to the American Society of Clinical Oncology guidelines, risk scores (Khorana Score, revised Khorana score, Vienna score and Protecht score) are helpful tools to identify patients at highest risk for VTE [79–81]. For further information on thromboprophylaxis in hospitalized patients with cancer, thromboprophylaxis in surgical patients with cancer, and treatment of thrombosis, please see Supplement Table S3 and accompanying text.

### Complications after new cancer drugs or cellular immunotherapy

Pharmacological and cellular treatment of cancer is changing dramatically not only with benefits for patient’s outcome and comfort, but also with new toxicity profiles. The vast majority of adverse events can be classified as mild or moderate but there are also some cases of severe and life-threatening complications requiring ICU admission. Diagnosis and management of severe side effects after (monoclonal or bispecific) antibody treatment, tyrosine kinase inhibitors, immune checkpoint inhibitors, and chimeric antigen receptor-modified (CAR-) T cells were summarized recently in a concise review by the iCHOP group [82].

### VAIA

#### Transfusions

In order to prevent the transfusion-associated graft versus host reaction (GvHR), all cellular blood products have to be irradiated with ≥ 30 Gy for patients with lymphatic diseases, autologous/allogeneic stem cell transplantation, and all patients treated with nucleoside analogues (fludarabine, cladribine) [83]. Generally, to prevent acute hemolysis, AB0-identic transfusions should be carried out. However, after allogeneic HSCT, in exceptional cases, it is possible to transfuse major or minor incompatible blood products according to the AB0 blood group of the patient and the donor as stated in Supplement Table S1 [84, 85]. Pooled platelet concentrates have a higher risk of immunization. Therefore, we recommend the transfusion of apheresis platelet concentrates [84].

#### Cytokine storm disease/Sepsis-like syndromes

Cancer patients are at risk to suffer from rare sepsis-like syndromes such as hemophagocytic lymphohistiocytosis (HLH), cytokine release syndrome (CRS), drug reaction with eosinophilia and systemic syndromes (DRESS), or capillary leak syndrome (CLS) [86–89]. Infections, malignancy itself, and drugs are major triggers for these sepsis-mimicking syndromes. Diagnostic vigilance for early recognition is essential to reduce mortality. To validate diagnosis, we recommend to include blood differential (cytopenia, eosinophilia), ferritin, soluble IL2R, fibrinogen, and triglycerides in addition to the routine ICU lab admission panel in cancer patients with sepsis or sepsis-like syndromes in whom there is no apparent focus of sepsis [90, 91] (see Table 6 for HLH diagnostic criteria). Withholding the assumed trigger drug and immediate search for an underlying malignancy or infection are pivotal. HLH patients require immediate immunosuppression with corticosteroids to prevent further progression of organ failure [92]. Early consultation with an HLH expert to stratify treatment according to the most likely trigger is recommended (www.hlh-registry.org).

**Table 6:** HLH diagnostic criteria [91]

HLH-2004 diagnostic criteria: ≥ 5 must be fulfilled
- Fever (≥ 38.3 °C)
- Splenomegaly
- Cytopenias in ≥ 2 lines (hb < 9 g/dL, plt < 100 /nL, neutrophils < 1.0/nL)
- Ferritin ≥ 500 μg/L
- Hypertriglyceridemia and/or hypofibrinogenemia (fasting triglycerides ≥ 265 mg/dL, fibrinogen < 1.5 g/L)
- Hemophagocytosis in bone marrow or spleen or lymph nodes
- Low or absent NK activity
- Soluble CD25 (soluble IL-2 receptor) ≥ 2400 U/mL

## Summary

During the last years, outcome of patients with malignant hematological or oncological diseases requiring intensive care treatment has clearly improved. From recently published data, we conclude that the severity of the acute illness is more important with regard to short-term survival than the underlying type and stage of malignancy. Patients receiving intensive treatment of their malignancy should be considered for ICU treatment as other severely ill patients without cancer. Centers should establish local admission criteria for critically ill cancer patients as well as standardized admission procedures, such as joint assessment by cancer specialists and intensivists, allowing identification of patients and standardized transfer to the ICU. In some patients, time-limited ICU trials with pre-defined do-not-escalate decisions (e.g., do-not-intubate or do-not-attempt-resuscitation) may be an adequate option until final decision. This implements that critically ill cancer patients should be referred to experienced intensive care units. We strongly recommend establishing a local structure with daily joint rounds of cancer specialists and intensivists as well as standard-operating procedures for the management of frequent medical conditions in critically ill cancer patients.

Furthermore, centers should organize continuous medical education of cancer specialists and intensivists. Finally, basic concepts of the care of critically ill cancer patients should be included into the curricula of both cancer specialists and intensivists.

In addition, the early integration of an interdisciplinary palliative care team is an urgently necessary measure.

In order to improve the management of critically ill cancer patients, knowledge of current practices in German-speaking countries is of utmost interest. To produce more robust data, participation of treating centers in central patient registries, e.g., the iCHOP registry (info@ichop.eu), is highly recommended.

## Electronic supplementary material

ESM 1 (DOCX 69 kb)

ESM 2 (DOCX 80.5 kb)
