# Supplementary material for: Consensus statement for cancer patients requiring intensive care support
Source: Ann Hematol. 2018 Apr 27;97(7):1271–82. doi: 10.1007/s00277-018-3312-y (PMC5973964; doi:10.1007/s00277-018-3312-y)
Supplement: Supplementary file 1 — (DOCX 69 kb) [file 277_2018_3312_MOESM1_ESM.docx]

Search strings

*(infection) AND (cancer) AND (icu)*

(infection) AND ((malignancy) OR (malignancies)) AND (icu)

(granulocyte-colony stimulating factor) AND (acute respiratory distress syndrome)

*hematologic* OR *malignancy* AND *icu* AND *anticoagulation*

*immunocompromised* AND *anticoagulation*

*Cancer* and *anticoagulation* and *ICU*

*Cancer* and *anticoagulation*

*thromboembolism* and *cancer* and *icu*

*cancer* and *bleeding* complication and *icu*

Section respiratory failure:

Cancer and patients and respiratory failure and/or ICU

Malignancy and respiratory failure and/or ICU

Hematologic and patients and respiratory failure and/or ICU

ARDS and malignancy

Malignancy and ICU

Allogeneic and stem and cell and transplantation and ICU

Allogeneic and stem and cell and transplantation and respiratory and failure

Allogeneic and stem and cell and transplantation and respiratory and ARDS

Allogeneic and stem and cell and transplantation and ICU and outcome

Mechanical ventilation and malignancy and outcome or survival

Mechanical ventilation and malignancy

Cancer and patients and ICU

Cancer patients and intensive and care and treatment

Cancer patients and intensive and care and treatment and palliative care

Hemophagocytosis

Cytokine-storm and cancer

Search history: until 23.2.2017
