# Supplementary material for: Consensus statement for cancer patients requiring intensive care support
Source: Ann Hematol. 2018 Apr 27;97(7):1271–82. doi: 10.1007/s00277-018-3312-y (PMC5973964; doi:10.1007/s00277-018-3312-y)
Supplement: Supplementary file 2 — (DOCX 80.5 kb) [file 277_2018_3312_MOESM2_ESM.docx]

Supplement

Specifics in the allogeneic stem cell transplant setting

1. Isolation procedures during transplantation

General precautions are necessary for treatment of allogeneic HSCT patients. To prevent nosocomial or health-care associated infections single room isolation under conditions of laminar airflow or positive pressure (high efficiency particulate air filter, HEPA) filtration, contact precautions and strict compliance with standardized infection control measures (i.e. hand disinfection) are mandatory. **Health-care workers with transmissible diseases (e.g. herpes, infectious gastroenteritis, respiratory tract infections) should be restrained from direct patient care** **(AII)** (Ullmann, Schmidt-Hieber et al. 2016).

1. Specific monitoring

We recommend a regular – if necessary daily - monitoring of drug trough levels for immunosuppressive agents (calcineurin inhibitors, i.e. cyclosporine or tacrolimus, or mTOR inhibitors, i.e. sirolimus or everolimus), and dose adjustment after consulting the transplant specialist **(A-III)**.

After allogeneic HSCT, patients at risk for cytomegalovirus (CMV) disease should be screened regularly, i.e. at least weekly, for pp65 antigenemia or by nucleic acid detection methods **(A-II)** (Ullmann, Schmidt-Hieber et al. 2016).

1. Typical complications after allogeneic HSCT
2. Acute graft-versus-host disease

Typical clinical signs or manifestations of this life-threatening condition are skin symptoms i.e. maculopapular skin rash, gastrointestinal tract (GI) symptoms including nausea and/or anorexia, watery diarrhea, GI tract bleeding, ileus, and stomach cramps as well as severe abdominal pain and cholestatic hyperbilirubinemia (Ferrara, Levine et al. 2009), and require immediate consultation of the transplant specialist. In such patients specific immunosuppressive and supportive therapy, including antibiotic, antifungal and antiviral therapy, are of utmost importance. With regards to detailed treatment recommendations (Zeiser 2015).

1. Hepatic veno-occlusive disease/sinusoidal obstruction syndrome

Veno-occlusive disease/sinusoidal obstruction syndrome (VOD/SOS) should be considered in any patient who has undergone allogeneic HSCT developing liver dysfunction. The diagnosis is based primarily on clinical diagnostic criteria. In case of unexplained occurrence of bilirubin ≥2 mg/dL (34 μmol/L) or painful hepatomegaly or weight gain >5% or ascites the diagnosis VOD/SOS has to be considered (Mohty, Malard et al. 2016) and, therefore, the transplant specialists have to contacted immediately.

1. Infections

Infectious complications are a major cause of morbidity and mortality in allogeneic HSCT patients (Tabbara, Zimmerman et al. 2002, Ullmann, Schmidt-Hieber et al. 2016). General recommendations on prophylaxis and treatment of infections in allogeneic HSCT patients are given in supplement table S1.

1. Thrombotic microangiopathy

Thrombotic microangiopathy (TMA) is a well-recognized complication occurring after allogeneic HSCT due to endothelial damage by the conditioning regimens (irradiation) and immunosuppressive agents (calcineurin inhibitors and sirolimus). The presence of hemolytic anemia with negative coombs test, thrombocytopenia and schistocytes in the blood smear lead to the diagnosis of TMA. Typical clinical features are renal dysfunction with hypertension and proteinuria and neurological abnormalities (Holbro and Passweg 2015). When TMA is suspected, transplant specialists and nephrologists have to be contacted.

**Table S1.** General recommendations for prophylaxis and treatment of infections in the allogeneic setting

| **Intention** | **Intervention** | **SoR/QoE** | **Comments** | **Reference** |
| --- | --- | --- | --- | --- |
| Prevent bacterial  Infections | Antibiotics before  reconstitution of neutrophils  Antibiotics after reconstitution  of neutrophils | BII_r_  DIII | Reduced incidence of  infection, but no impact  on mortality  Without chronic GvHD | (93)  (53) |
| Primary prevention *Pneumo-*  *cystis* pneumonia | Trimethoprim/sulfamethoxazole  after alloHSCT | AII_t_ | Up to 6 months after alloHSCT, longer in case of active GvHD or ongoing immunosuppression | (53) |
| Primary prevention toxo-  plasmosis | Antiparasitics after alloHSCT | DIII | Trimethoprim/sulfamethoxazole  for *Pneumocystis* prevention  is also effective against  toxoplasmosis | (53) |
| Secondary prevention toxo-  plasmosis | Pyrimethamine/sulfadiazine  after alloHSCT | AII_t_ | ≥3 months after successful  therapy | (53) |
| Prevent mold infection | Posaconazole | BII_t_ | Until day +100 without GvHD | (53) |
| Prevent invasive aspergillosis | Posaconazole | AI | With active GvHD  Improved survival due to  invasive fungal infections | (53) |
| Prevent CMV infection/  Disease | Ganciclovir, valganciclovir or  foscarnet | AI | Pre-emptive strategy  recommended over  prophylaxis/treatment | (53) |
| Treat fever/sepsis in  Neutropenia | Meropenem, imipenem/  cilastatin or piperacillin/  tazobactam | AIII | Combination with amino-  glycosides in septic shock/  severe sepsis may be  considered (BIII) | (59) |
| Treat invasive aspergillosis | Voriconazole | AI |  | (50) |
| Treat CMV disease | Ganciclovir | AI |  | (94) |

**Note.** SoR, strength of a recommendation; QoE, quality of evidence; GvHD, graft-versus-host disease; alloHSCT, allogeneic hematopoietic stem cell transplantation; CMV, cytomegalovirus

**Table S2: Anticoagulation in hematologic and oncologic patients**

|  | Clinical situation/intervention | SoR | QoE | Reference | Comments |
| --- | --- | --- | --- | --- | --- |
| 1. Q: Risk factors | Risk factors (ICU) |  |  |  | No data available |
|  | Risk factors (not ICU!)  Kind of cancer; Ctx |  | II (retrospective, observational cohort study) 2004-2009; n= 17.284 | (95) | highest rates of VTE (ambulatory): Pancreatic 19,2%, stomach 15,8%, lung 13,9%, ovarian 11%, colorectal 10,6%, bladder 8,2% greatest risk for VTE within 3 months of the initial diagnosis |
|  | Risk factors (not ICU!)  Kind of cancer |  | II (Population-Based Study) 1988-2000; n=1417 | (96) | highest rates of VTE: brain 47,3% tp head neck cancer 4,1% |
|  | Risk factors (not ICU!)  Kind of cancer |  | II (population-based, case-control) 1999-2002; n=3220 | (97) | Highest rate of VTE: hematological, lung cancer, gastrointestinal cancer. VTE within first few month |
|  | Risk factors (not ICU!)  NHL/ HL |  | II (prospective cohort study) 2008-2010; n= 686 | (98) | newly diagnosed Hodgkin's lymphoma (HL) and non-Hodgkin's lymphoma (NHL): NHL 8%, HL 6,7%. TVT 61,1%, PE 38,9%. |
|  | Risk factors  (not ICU!) Ctx |  | II (population-based, nested case-control study) n=625 | (99) | Malignant Ctx 6,53 increased risk for DCT |
|  | Risk factor (not ICU!) CVC |  | II (population-based, nested case-control study) n=625 | (99) | CVC 5,55 increased risk |
|  | Risk factor (not ICU!) CVC and acute leukemia |  | II (retrospective study) n=299 ALL/ 996 AML | (100) | VTE ALL 20,7%, AML 18,6%. High risk for VTE and PE was CVC associated |
|  | Risk factor (not ICU!) Long-term CVC |  | II (review article) | (101) | Incidence for DVT for long term CVC: 0,3-28,3%. For PE 15-25%. |
| 2. Q: Risk assessment | Risk assessment (not ICU!) | A | II (observational study) n=2701 | (69) | Khorana Score |
|  | Risk assessment (not ICU!) | A | II (observational study) n=2170 | (70) | Revised Korana Score |
|  | Risk assessment (not ICU!) | A | II (study population) 2003-2008; n=819 | (102) | Vienna VTE prediction score |
|  | Risk assessment (not ICU!) |  | II | (71) | Protecht Score |
| 3. Q: Prophylaxis | General prophylaxis (not ICU) | A | I (RCT) | (71) | Comparison of Khorana Score and Protecht score and RCT prophylaxis with Nadroparin vs. placebo  Khorana score ≥3 NNT 15; Khorana score <3 NNT 77 with Nandroparin |
|  | General prophylaxis (not ICU) | A | I (RCT) n=3212 | (103) | SAVE-ONCO study; Semuloparin 1,2% versus 3,4% placebo; no serious bleeding, similar thrombocytopenia in both groups |
|  | Hospitalized patients prophylaxis (not ICU) | C | II (Meta-analysis of RCT) | (104) | 3 RCT with TVT as primary outcome and type of cancer. Risk for TVT 0,91 among hospitalized patients with cancer who receiving thromboprophylaxis. 🡪 lack of evidence to support thromboprophylaxis in hospitalized medical patients with cancer |
|  | Hospitalized patients prophylaxis (not ICU) |  | II (ASCO guidelines; review of RCT,  n= 53 studies | (105) | ASCO Guidelines 2015 revised:  thromboprophylaxis throughout hospitalization; |

Thromboprophylaxis in Hospitalized Patients with Cancer

1. Hospitalized patients with active malignancy and acute medical illness or reduced mobility should receive thromboprophylaxis (either pharmacologic or mechanical) in the absence of contraindications.

2. In the absence of additional risk factors, hospitalized patients with cancer may be considered for VTE prophylaxis.

3. There is currently insufficient evidence to support VTE prophylaxis in patients admitted for minor procedures or a short infusion of chemotherapy.

Thromboprophylaxis in Surgical Patients with Cancer

1. All patients with malignancy undergoing major surgical intervention should be considered for thromboprophylaxis (either pharmacologic or mechanical) unless contraindications exist. VTE prophylaxis should start preoperatively.

2. Moreover, it is recommended that pharmacologic thromboprophylaxis should be continued for 7–10 days in all patients, with the exception of patients undergoing major abdominal or pelvic surgery for cancer who have additional morbidity, in whom VTE prophylaxis should be continued for up to 4 weeks.

The major concerns for thromboprophylaxis are the contraindications. 31.9% had relative contraindications to anticoagulation. Of these patients, the most common contraindication was thrombocytopenia (65.2%), followed by active hemorrhage (17.4%) (106).

Treatment of thrombosis

Critically ill cancer patient on ICU with VTE need additional unique management considerations. The goal is to achieve optimal risk-benefit ratio for each individual patient. Anticoagulation is appropriate for most patients with VTE/ PE until thrombocytes count is less than 50.000/µl. There is no evidence for an anticoagulation at platelets below 50.000/µl. An individual decision in low platelet patients is required.

Also, acute thrombolysis in life threatening situations induced by cancer requires case by case decisions.

**Table S3.** Recommendations for transfusions in the allogeneic setting from the beginning of the conditioning (74, 75)

| **Patient** | **Donor** | **Red cells** | **Platelets**  **1^st^ choice** | **Platelets**  **2^nd^ choice** | **Plasma** |
| --- | --- | --- | --- | --- | --- |
| **Major incompatible** | | | | | |
| 0 | A | 0 | A | AB, B, 0 | A, AB |
| 0 | B | 0 | B | AB, A, 0 | B, AB |
| 0 | AB | 0 | AB | A, B, 0 | AB |
| A | AB | A | AB | A, B, 0 | AB |
| B | AB | B | AB | B, A, 0 | AB |
| **Minor incompatible** | | | | | |
| A | 0 | 0 | A | AB, B, 0 | A, AB |
| B | 0 | 0 | B | AB, A, 0 | B, AB |
| AB | 0 | 0 | AB | A, B, 0 | AB |
| AB | A | A | AB | A, B, 0 | AB |
| AB | B | B | AB | B, A, 0 | AB |
| **Major and minor incompatible** | | | | | |
| A | B | 0 | AB | A, B, 0 | AB |
| B | A | 0 | AB | B, A, 0 | AB |

Additional Literature

93.       Kimura S, Akahoshi Y, Nakano H, Ugai T, Wada H, Yamasaki R, et al. Antibiotic prophylaxis in hematopoietic stem cell transplantation. A meta-analysis of randomized controlled trials. J Infect. 2014;69(1):13-25.

94.       Einsele H, Bertz H, Beyer J, Kiehl MG, Runde V, Kolb HJ, et al. Infectious complications after allogeneic stem cell transplantation: epidemiology and interventional therapy strategies--guidelines of the Infectious Diseases Working Party (AGIHO) of the German Society of Hematology and Oncology (DGHO). Ann Hematol. 2003;82 Suppl 2:S175-85.

95.       Khorana AA, Dalal M, Lin J, Connolly GC**.** Incidence and predictors of venous thromboembolism (VTE) among ambulatory high-risk cancer patients undergoing chemotherapy in the United States. Cancer. 2013;119(3):648-55.

96.       Petterson TM, Marks RS, Ashrani AA, Bailey KR, Heit JA**.** Risk of site-specific cancer in incident venous thromboembolism: a population-based study. Thromb Res. 2015;135(3):472-8.

97.       Blom JW, Doggen CJ, Osanto S, Rosendaal FR**.** Malignancies, prothrombotic mutations, and the risk of venous thrombosis. JAMA. 2005;293(6):715-22.

98.       Park LC, Woo SY, Kim S, Jeon H, Ko YH, Kim SJ, et al. Incidence, risk factors and clinical features of venous thromboembolism in newly diagnosed lymphoma patients: results from a prospective cohort study with Asian population. Thromb Res. 2012;130(3):e6-12.

99.       Heit JA, Silverstein MD, Mohr DN, Petterson TM, O'Fallon WM, Melton LJ, 3rd**.** Risk factors for deep vein thrombosis and pulmonary embolism: a population-based case-control study. Arch Intern Med. 2000;160(6):809-15.

100.     Vu K, Luong NV, Hubbard J, Zalpour A, Faderl S, Thomas DA, et al. A retrospective study of venous thromboembolism in acute leukemia patients treated at the University of Texas MD Anderson Cancer Center. Cancer Med. 2015;4(1):27-35.

101.     Verso M, Agnelli G**.** Venous thromboembolism associated with long-term use of central venous catheters in cancer patients. J Clin Oncol. 2003;21(19):3665-75.

102.     Ay C, Dunkler D, Marosi C, Chiriac AL, Vormittag R, Simanek R, et al. Prediction of venous thromboembolism in cancer patients. Blood. 2010;116(24):5377-82.

103.     Agnelli G, George DJ, Kakkar AK, Fisher W, Lassen MR, Mismetti P, et al. Semuloparin for thromboprophylaxis in patients receiving chemotherapy for cancer. N Engl J Med. 2012;366(7):601-9.

104.     Carrier MK, AA; Moretto, P; Le Gal, G; Karp, R; Zwicker, JI**.** Lack of Evidence to Support Thromboprophylaxis in Hospitalized Medical Patients with Cancer. . American Journal of Medicine. 2014;127:5.

105.     Lyman GH, Bohlke K, Khorana AA, Kuderer NM, Lee AY, Arcelus JI, et al. Venous thromboembolism prophylaxis and treatment in patients with cancer: american society of clinical oncology clinical practice guideline update 2014. J Clin Oncol. 2015;33(6):654-6.

106.     Zwicker JI, Rojan A, Campigotto F, Rehman N, Funches R, Connolly G, et al. Pattern of frequent but nontargeted pharmacologic thromboprophylaxis for hospitalized patients with cancer at academic medical centers: a prospective, cross-sectional, multicenter study. J Clin Oncol. 2014;32(17):1792-6
